# 'SOSORT consensus paper on brace action: TLSO biomechanics of correction (investigating the rationale for force vector selection)'

**DOI:** 10.1186/1748-7161-1-11

**Published:** 2006-07-20

**Authors:** M Rigo, S Negrini, HR Weiss, TB Grivas, T Maruyama, T Kotwicki, the members of SOSORT

**Affiliations:** 1 E.Salvá Institute. Vía Augusta 185. 08021 Barcelona, Spain; 2 ISICO (Italian Scientific Spine Institute). Studio Paolo Sibilla, Via Carlo Crivelli 20, 20122 Milan, Italy; 3 Asklepios Katharina Schroth Spinal Deformities Rehabilitation Centre. Korczacstr.2, D-55566 Bad Sobernheim, Germany; 4 Scoliosis clinic, Orthopaedic Department, Thriasio General Hospital, G. Genimata Avenue, Magula 19660, Greece; 5 Department of Orthopaedic Surgery, Teyko University School of Medicine 2-11-1 Kaga, Itabashi-ku, Tokyo, 173-8605, Japan; 6 Department of Paediatric Orthopaedics. Karol Marcinkowski University of Medical Sciences. ul. 28 Czerwca 1956 roku nr 135, 61-545 Poznan, Poland

## Abstract

**Background:**

The effectiveness of orthotic treatment continues to be controversial in international medical literature due to differences in the reported results and conclusions of various studies. Heterogeneity of the samples has been suggested as a reason for conflicting results. Besides the obvious theoretical differences between the brace concepts, the variability in the technical factors can also explain the contradictory results between same brace types. This paper will investigate the degree of variability among responses of scoliosis specialists from the Brace Study Ground of the International Society on Scoliosis Orthopedic and Rehabilitation Treatment SOSORT. Ultimately, this information could be a foundation for establishing a consensus and framework for future prospective controlled studies.

**Methods:**

A preliminary questionnaire on the topic of 'brace action' relative to the theory of three-dimensional scoliosis correction and brace treatment was developed and circulated to specialists interested in the conservative treatment of adolescent idiopathic scoliosis. A particular case was presented (main thoracic curve with minor lumbar). Several key points emerged and were used to develop a second questionnaire which was discussed and full filed after the SOSORT consensus meeting (Milano, Italy, January 2005).

**Results:**

Twenty-one questionnaires were completed. The Chêneau brace was the most frequently recommended. The importance of the three point system mechanism was stressed. Options about proper pad placement on the thoracic convexity were divided 50% for the pad reaching or involving the apical vertebra and 50% for the pad acting caudal to the apical vertebra. There was agreement about the direction of the vector force, 85% selecting a 'dorso lateral to ventro medial' direction but about the shape of the pad to produce such a force. Principles related to three-dimensional correction achieved high consensus (80%–85%), but suggested methods of correction were quite diverse.

**Conclusion:**

This study reveals that among participating SOSORT specialists there continues to be a strongly held and conflicting if not a contentious opinion regarding brace design and treatment. If the goal of a 'treatment consensus' is realistic and achievable, significantly more effort will be required to reconcile these differences.

## Background

Orthotic bracing is the most common non-surgical treatment for Adolescent Idiopathic Scoliosis (AIS), either alone or in combination with exercises. In spite of some negative reports [1-3], brace treatment has been shown to change the natural history of AIS [4] and reduce the incidence of surgery [5-7]. However, the mechanism of action by which braces prevent curve progression, is not well understood.

Generally speaking, bracing should unload the growth plates of the apical vertebral bodies on the concavity. Stokes has shown that an imposed vertebral deformity can be corrected by reversing the load used to create it [8]. Therefore, the principles of the Hueter Volkmann law should be applicable to the correction of a scoliotic curve when there is sufficient residual growth. Nevertheless, evidence demonstrating that braces can affect the vertebral growth in spinal deformities is limited.

Design principles for many braces are contradictory and based on dated concepts, proposed and tested decades ago when the three-dimensional (3D) nature of AIS was rarely considered or incorporated into the brace design. The proper biomechanical principles for orthotic correction should apply derotational forces that correct in the coronal and axial plane in addition to producing normal spinal alignment in the sagittal plane [9].

In spite of numerous papers reporting on brace biomechanics [10-28], no principle, except perhaps 'the three point system', seems to be universally accepted. Many clinicians seem to fit braces empirically rather than using "curve-specific" biomechanical 3D models.

This paper will investigate the degree of variability among responses of scoliosis specialists from the Brace Study of the International Society on Scoliosis Orthopedic and Rehabilitation Treatment -SOSORT- (described below) regarding brace action and the selection of force vectors to effect optimal curve correction. Each participant was given the same patient scenario and curve pattern and was asked to complete a questionnaire indicating their treatment approach. Ultimately this information could be a foundation for establishing a consensus and framework for future prospective controlled studies.

### Foundation for consensus

The formation of the SOSORT was a significant accomplishment of the Conference on Conservative management of Spinal Deformities, held January 2004 in Barcelona, Spain [29]. At this meeting, participants agreed on the need for a prospective, multi-center, multi-national, controlled study using more specific bracing techniques that those reported with the Boston brace. They also agreed that a comprehensive patient outcome was a more important measure of success than the Cobb angle alone and this outcome should be based on assessment variables such as vertebral rotation, sagittal alignment, appearance of deformity, functional abilities and quality of life.

However, there was obvious disagreement, between different schools of thought, about how braces should correct a particular scoliosis pattern. Intensive discussion on brace action was postponed to future meetings, as participants sought a consensus before designing a prospective controlled study.

## Methods

Following the formation of SOSORT in Barcelona, January 2004, a preliminary questionnaire was developed and circulated to all attendees and many specialists interested in the conservative treatment of adolescent idiopathic scoliosis. The simple questionnaire solicited their opinion on the topic of "brace action "where to push" and "why"" relative to the theory of 3-D scoliosis correction and brace treatment.

**Figure 1:**
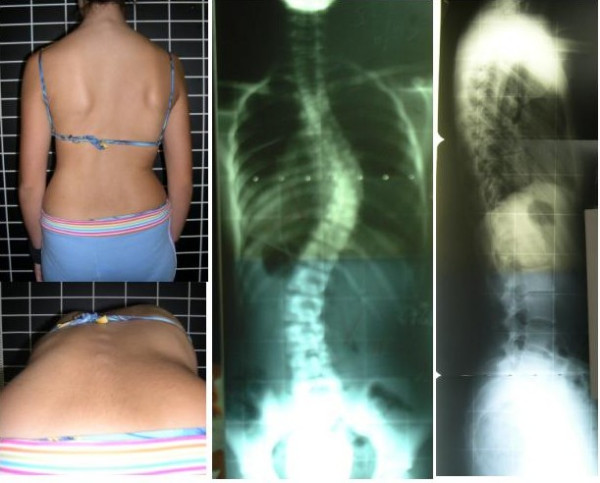
Patient information included dorsal and forward bending photographs, as well as AP and latero-lateral radiographs.

Originally, two clinical cases were presented; only one was selected for use in this study. Complete Patient information including dorsal, ventral, lateral and forward bending photographs, as well as AP and lateral radiographs were provided (Figure 1). While the results of the questionnaire where not useful for developing a statistical study, they provided general opinions and treatment ideas. Nevertheless, a summary document of results was sent to all participants prior to the "SOSORT" consensus meeting in Milan, January 2005, where it was discussed in detail. From the consensus discussion and an exhaustive analysis of the preliminary questionnaire, several key points of interest emerged and became the impetus for developing a second questionnaire and the focus of this study. These were: the type of brace, the level and direction of the force vector necessary to correct the convexity of the main thoracic curve, the shape of the pad pushing the dorsal rib hump, the three point system principle in the frontal plane, the necessity for a 'ventral to dorsal' force on the ventral rib hump, derotation of the main thoracic curve, normalization of the sagittal profile, the level and direction of the force vector to correct the lumbar curve, the necessity for abdominal pressure and the design of the pelvic section.

## Results

Twenty-one questionnaires were completed and returned for inclusion in this study. All but two of the responses recommended using a TLSO in treating the specified case. Of the two other responses, one did not answer this question and the other selected a Milwaukee brace. The most consistent selection was The Chêneau brace (13 times) and its variations. In addition, the Lyon brace was chosen three times and the modified Boston was chosen once.

**Figure 2:**
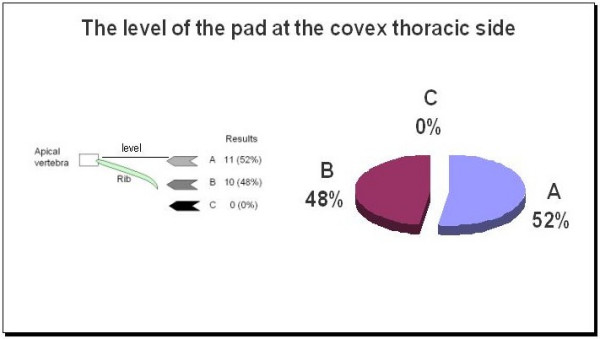
The vector force level to correct the thoracic convexity.

**Figure 3:**
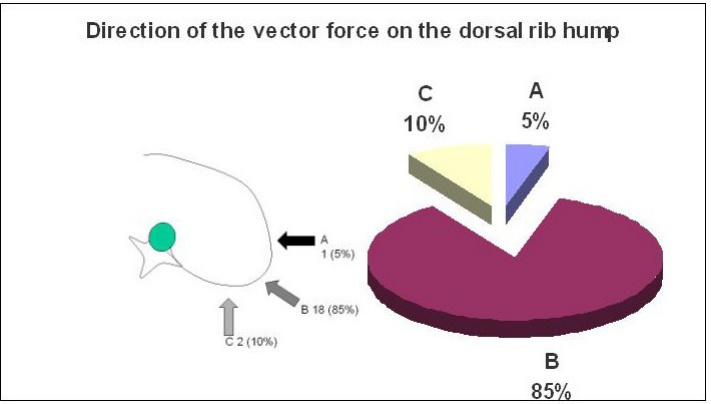
The direction of the vector force correcting the dorsal rib hump.

**Figure 4:**
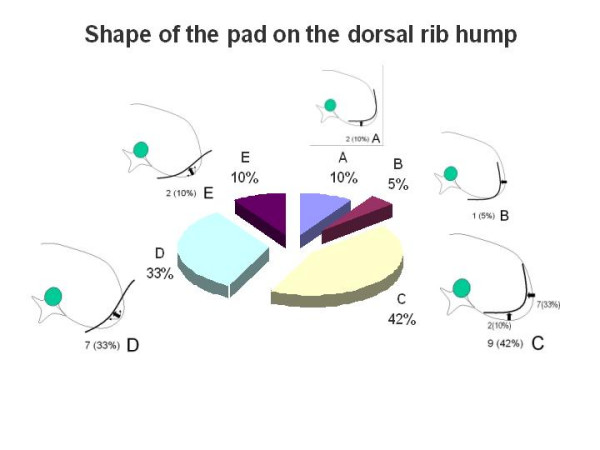
The shape of the pad correcting the dorsal rib hump.

The responses were almost evenly divided regarding the proper placement of the thoracic pad on the convexity of the thoracic curve. Eleven selected pad placement at the level of the apical vertebra and ten placed it below the apical vertebra but at the apical rib. Figure 2 shows the picture used in the questionnaire and the proportion for each answer. There was a high percentage of agreement about the orientation of the vector force necessary to correct the convexity of the main thoracic curve. Most specialists defined it as an oblique vector with a 'dorso-lateral to ventro-medial' direction as seen in Figure 3. However, there was significant disparity in the responses regarding the most appropriate shape of the thoracic pad acting on the dorsal rib hump. Figure 4 shows the different proposed shapes and the number of times each type was selected.

**Figure 5:**
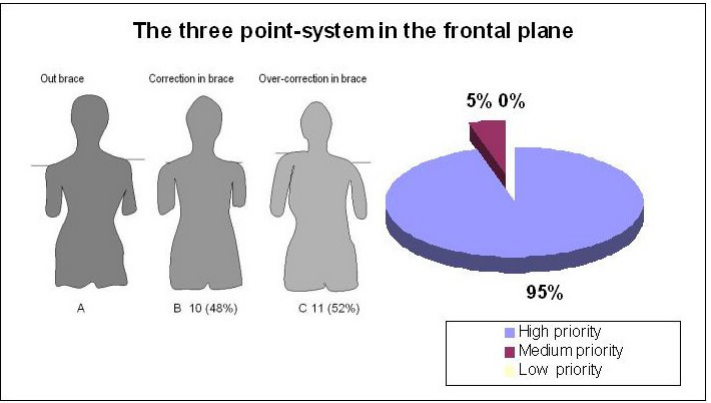
The importance of the three point system to correct in thoracic main curvature in the frontal plane.

When asked about the importance of the 'three-point system' principle in the frontal plane, nearly everyone agreed that correcting the scoliotic curve in the frontal plane was a high priority (figure 5). In the case of a right convex thoracic scoliosis, the principle would be properly applied using forces on the left lumbar convexity, the right thoracic convexity and the left upper thoracic. However, the specialists were divided almost 50% in their preference when asked about the three-point system correcting or over-correcting the shoulder imbalance as showed in figure 5.

**Figure 6:**
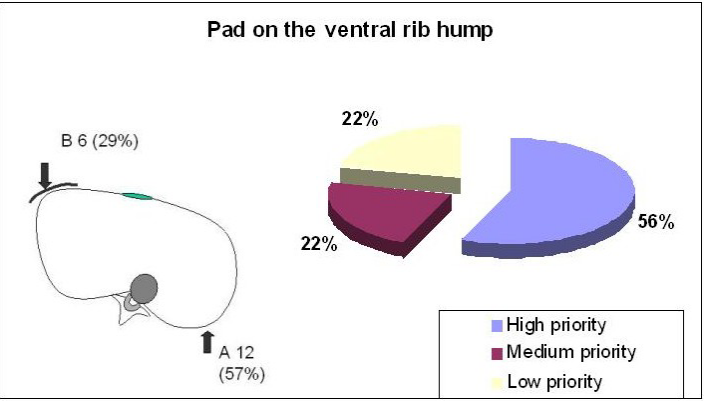
The pad correcting the ventral rib hump.

**Figure 7:**
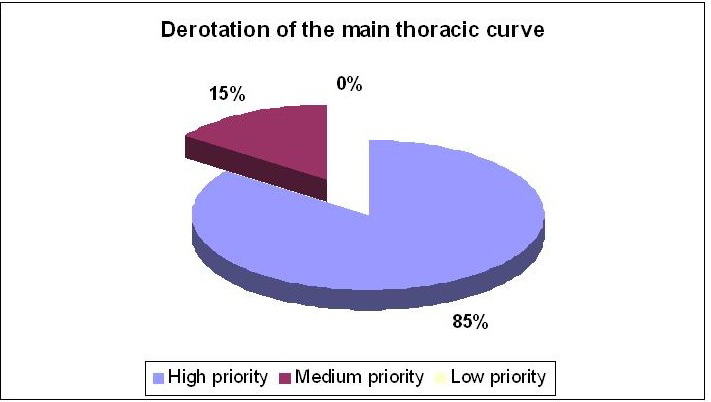
Derotation of the main thoracic curve.

Addressing the necessity of a 'ventral to dorsal' force on the concave thoracic side by pushing with a pad on the ventral rib hump, fifty-six percent of the responses gave this principle high priority, 22% medium priority and 22% low priority (Figure 6). The questionnaire offered two choices, reflecting different definitions of this principle. Accordingly, 57% selected option A "the pad on the ventral rib hump acts as a counter-force to the pad on the dorsal rib hump in order to form a pair of forces". Option B was chosen by 29%, defining the ventral pad as the main pad for derotation (pair of forces) as well as for the reconstruction of the normal thoracic kyphosis. Similarly, the derotation of the main thoracic curve was considered a high priority by 85% of the experts and a medium priority by 15% (Figure 7).

**Figure 8:**
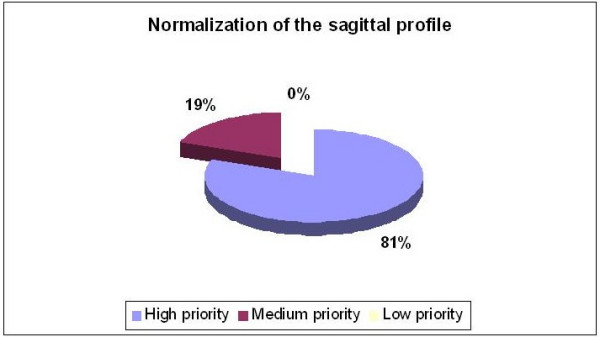
Normalization of the sagittal configuration.

Reconstruction or normalization of the sagittal profile was considered an important concept (Figure 8). All the specialists considered this principle to be of high (81%) or medium (19%) priority.

**Figure 9:**
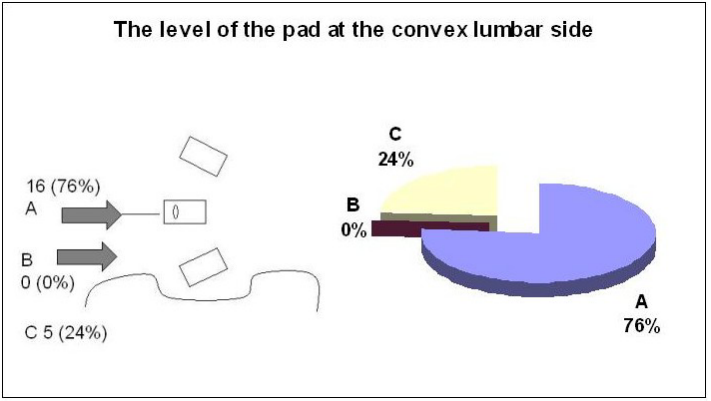
The level of the pad correcting the lumbar convexity.

**Figure 10:**
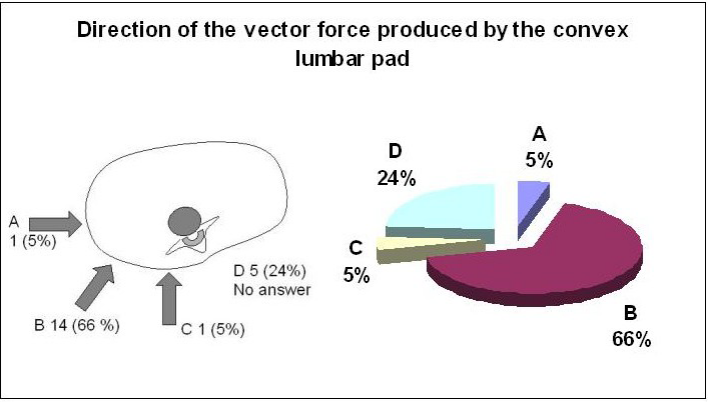
The direction of the vector force correcting the lumbar convexity.

When asked to identify which vector force they would use to correct the lumbar convexity, 76% of the group recommended a force reaching the apical vertebra of the lumbar curve (Figure 9). There was no response from 24% and no one recommended pushing caudal to the apex. As to the appropriate placement of the force-vector on the convexity of the lumbar curve, 66% percent recommended to push from dorso-lateral to ventro-medial. Five percent selected either a lateral or posterior only placement and 24% percent did not answer(Figure 10).

**Figure 11:**
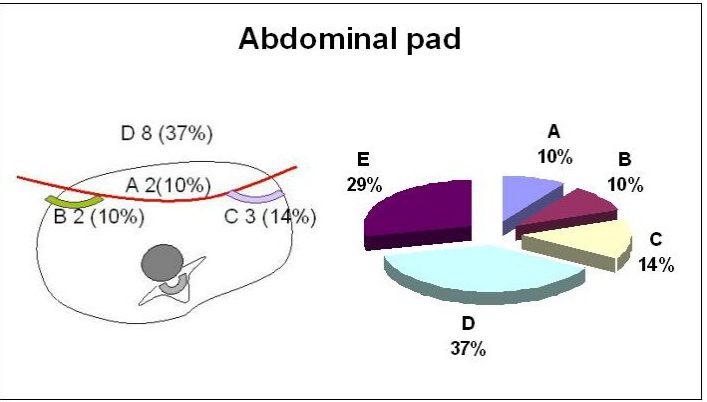
The abdominal pressure.

When asked about the necessity for a ventral pad on the abdominal area, 37% percent of the experts did not recommend pushing ventrally (figure 11). Ten percent pushed symmetrically, while another 10% pushed with an asymmetric pad on the left. The remaining 14% pushed on the right. The question was proposed using text only; the picture in figure 11 was added later in order to graphically represent the distribution of responses.

**Figure 12:**
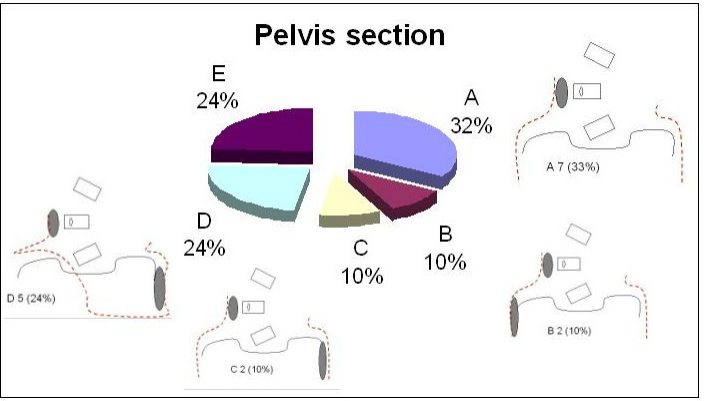
The design of the pelvis section.

The design of the pelvic section was the last area of focus. Figure 12 shows four different and in some cases contradictory designs. The most frequently chosen, 32%, was a bilaterally closed and symmetric pelvis. Twenty-four percent selected a semi-open, asymmetrical pelvic section, pushing on the right side and leaving room on the left side. Twenty percent of the responses were evenly split between the remaining two designs. Twenty-four percent of the experts did not answer the question.

## Discussion

The results of this study show the diversity of ideas and personal interpretations about the biomechanics of correction, brace design and treatment application in the proposed case. There was a wide range of treatment responses for this adolescent girl diagnosed with a classic idiopathic pattern of main right thoracic and minor left lumbar curvatures. Consensus was reached for three main biomechanical principles, at least from a theoretical point of view.

First, nearly everyone agreed on the importance of using the 'three point system' to correct the main thoracic curvature in the frontal plane. Principles related to 3D correction like 'derotation' and 'normalization of the sagittal profile', achieved greater consensus after the meeting (80%–85%) rather than before (50%). However, the suggested methods of correction were quite diverse.

When participants were asked which brace they preferred for this particular case, the Chêneau brace was the most frequently recommended. However, the variability in answers to specific questions on the biomechanics of correction indicates there are differing treatment methodologies. Consequently, the use of a particular name for a custom made brace, like the Chêneau, is no guaranty that there is a consistent standard in design or treatment.

A high thoracic extension on the concave thoracic side acts as the upper oppositional counter pad of the 'three point system' in this case. However, its effectiveness is enhanced or minimized by the pad placement and the direction of the force vector pushing the thoracic convexity. Over-correction of the shoulder level by using a left high thoracic counter pad was recommended by 50% of respondents, while the other 50% preferred a neutral, balanced alignment with no overcorrection. Several biomechanical studies have supported the use of the" three point system". Jonasson-Rajala et al [10] and later Perie et al [26] demonstrated that a high thoracic – axillary counter pad more effectively reduced both thoracic and lumbar curves rather than a lumbar pad combined with a low thoracic pad alone. Initial in brace correction, or primary correction, together with compliance, have been shown to be the most important factors predicting success of treatment [30]. Thus, calculating the primary correction obtained could test the effectiveness of the 'three point system' applied for any particular brace. Further studies are necessary comparing correction and over-correction of the shoulder imbalance in order to recommend one or the other method.

Opinions about proper pad placement on the thoracic convexity were evenly split. Fifty percent opted for the pad reaching or involving the apical vertebra and the other 50% for the pad acting caudal to the apical vertebra but including the apical rib. However, 85% of respondents agreed upon the direction of the force vector, selecting the 'dorso lateral to ventro medial' direction. Both mechanisms, the placement of the pad and the direction of the force vector are important for the 'three points system' principle as well as for 3D correction principles.

The necessary combined forces to correct a thoracic scoliosis in 3D has been shown by Gignac D et al, [13] in a simulation of two new approaches comparing their results to the Boston brace. Different forces were applied at the thoracic apex level and the posterior displacement of the rib hump was blocked. An oblique force oriented 45°c degrees with respect to the frontal plane was added at the lumbar apex. The main force at thoracic level was applied with a dorsal direction on the ventral rib hump. A second main force pushed on the convexity with a latero medial direction. The suggested mechanism was able to reduce the Cobb angle in the frontal plane while maintaining the normal physiological curvatures in the sagittal plane, reducing axial rotation and rib hump. However, this new treatment approach must be personalized for each patient and still requires clinical evaluation.

In this consensus study the direction of the force vector pushing the thoracic convexity, which has been recommended for the majority of the participants, includes the lateral to medial direction. However it also includes a dorsal to ventral force, which would reduce the thoracic kyphosis. Designing a ventral pad pushing ventral to dorsal on the concave thoracic side could prevent this. This pad, oriented close to the frontal plane, in combination with a secondary dorsal pad on the convex side, oriented close to the sagittal plane, would be the suggested 3D mechanism. It effectively creates a 'pair of forces' to derotate in the axial plane and a coupling mechanism to increase the thoracic kyphosis.

Theoretically, this is the biomechanical principle of the Chêneau brace, the most recommended brace in this study. Despite this technical knowledge and preference for this specific brace, and though many of the specialists talk '3D correction', ironically, very few of them indicated an effective application of it. This could be the reason why the Chêneau brace has been reported to avoid thoracic flat back, for some authors [22,31,32], while for some others it increases the thoracic lordosis [19]. This necessitates further study to clearly demonstrate that a particular brace can achieve an 'in vivo' 3D correction of a scoliotic curve.

Responses to the questions about abdominal pressure and the design of the pelvic section show not only a lack of agreement but contradictory design principles. Perhaps indicating that these are not essential elements in stabilizing the curvature in the proposed case or are concepts requiring further exploration and discussion before agreement is reached.

Although, all the SOSORT participants are recognized specialists in the treatment of scoliosis, having extensive knowledge of medical literature and significant clinical experience, many international opinions are missing from this study. Their inclusion would have increased the sample size and may have affected the outcome. Nevertheless, the objective of this study was not to establish new treatment guidelines as much as measure the level of consensus regarding current theoretical and treatment principles.

## Conclusion

Through open discussion and completion of specific questionnaires a 'treatment consensus' was sought; a foundational consensus, essential for creating a future, prospective, multicenter, multinational study on bracing effectiveness. While the interactive process and discussions were constructive and some points of agreement were identified...was there sufficient progress towards an essential consensus?. Unfortunately, this study reveals that among participating SOSORT specialists there continues to be a strongly held and conflicting if not contentious opinion regarding TLSO design and treatment. If the goal of a 'treatment consensus' is realistic and achievable, significantly effort will be required to reconcile these differences.

## Competing interests

The author(s) declare that they have no competing interests.

## Authors' contributions

MR, SN, HRW, TBG, TM, TK conceived of the study, and participated in its design and coordination and helped to draft the manuscript.

SN also performed the statistical analysis.
